# Multidisciplinary consensus on cancer management during pregnancy

**DOI:** 10.1007/s12094-020-02491-8

**Published:** 2020-11-16

**Authors:** A. Cubillo, S. Morales, E. Goñi, F. Matute, J. L. Muñoz, D. Pérez-Díaz, J. de Santiago, Á. Rodríguez-Lescure

**Affiliations:** 1 Department of Medical Oncology, Centro Integral Oncológico Clara Campal, Hospital Universitario HM Sanchinarro, Sociedad Española de Oncología Médica (SEOM), Madrid, Spain; 2 Gynecologic Oncology Unit, Hospital Universitario Infanta Leonor, Sociedad Española de Ginecología y Obstetricia (SEGO), Madrid, Spain; 3 Nuclear Medicine Service, Complejo Hospitalario de Navarra, Sociedad Española de Medicina Nuclear e Imagen Molecular (SEMNIM), Pamplona, Spain; 4 Radiology Department, Hospital Clínico San Carlos, Sociedad Española de Radiología Médica (SERAM), Madrid, Spain; 5 Oncological Radiotherapy Service, Hospital Universitario de Badajoz, Sociedad Española de Oncología Radioterápica (SEOR), Badajoz, Spain; 6 General Surgery Service, Hospital Clínico San Carlos, Asociación Española de Cirujanos (AEC), Madrid, Spain; 7 Gynecologic Oncology Unit, MD Anderson Cancer Center, Sociedad Española de Ginecología y Obstetricia (SEGO), Madrid, Spain; 8 Department of Medical Oncology, Hospital General Universitario de Elche, Sociedad Española de Oncología Médica (SEOM), Elche, Spain; 9 Grupo Español de Investigación en Cáncer de mama (GEICAM), Madrid, Spain

**Keywords:** Antineoplastic agents, Child development, Diagnosis, Follow-up, Prenatal care, Radiotherapy, Surgery, Systemic therapies

## Abstract

Cancer during pregnancy is a challenge for multi- and interdisciplinary collaboration due to the diagnostic, prognostic and therapeutic implications, the need for an integrated harmonization of medical action for the pregnant patient and the embryo or foetus and the characteristics of each gestational period, which will determine the protocol to be proposed and its limitations. For this reason, a group of experts appointed by participating scientific societies, which includes the Spanish Society of Medical Oncology (*Sociedad Española de Oncología Médica*—SEOM), the Spanish Association of Surgeons (*Asociación Española de Cirujanos*—AEC), the Spanish Society of Gynaecology and Obstetrics (*Sociedad Española de Ginecología y Obstetricia*—SEGO), the Spanish Society of Nuclear Medicine and Molecular Imaging (*Sociedad Española de Medicina Nuclear e Imagen Molecular*—SEMNIM), the Spanish Society of Oncological Radiotherapy (*Sociedad Española de Oncología Radioterápica*—SEOR) and the Spanish Society of Medical Radiology (*Sociedad Española de Radiología Médica*—SERAM), have worked together to establish consensus recommendations that allow the harmonization of management and ultimately the optimization of the healthcare of pregnant patients with cancer. When cancer is detected in a pregnant woman, the week of gestation in which the diagnosis is made must be considered, as well as the characteristics of the tumour. It is strongly recommended that a multidisciplinary team assesses the situation and guides the patient and her family during the informing, diagnosis and treatment process. Likewise, the foetus should be monitored and managed by specialized obstetricians who are part of a multidisciplinary cancer committee.

## Introduction

Gestational cancer is defined as cancer that occurs during pregnancy or during the year after delivery. In European countries, the incidence of gestational cancer has remained stable during the twenty-first century, after an annual increase of 1.5% in the final decades of the last century, with an incidence of 90-120 cases per 100,000 pregnancies [1, 2]. Among them, breast cancer ranks first by frequency, accounting for a third of all incident cases. After breast cancer, then thyroid cancer, cervical cancer, lymphomas and melanoma follow, ranked from high to low incidence (Table 1) [2, 3]. The delay in the age of pregnancy in the West carries an increased risk of gestational cancer, being 4 times higher in women over the age of 40 years compared to those under 30 years of age [2].

**Table 1:** Incidence of different types of cancer during pregnancy

Tumour	Frequency	
	Cases/10^5^ pregnancies	Cases/10^5^ births
Hodgkin lymphoma [82]		8.1
Breast cancer [83]		6.5
Non-Hodgkin lymphoma [84]		5.4
Melanoma [12]	2.8–5.0	
Cervical cancer [37]	1.4–4.6	
Ovarian cancer [37]	0.2–3.8	
Colorectal cancer [40]	2.0	

Cancer during pregnancy is a challenge for multi- and interdisciplinary collaboration [3] because of the diagnostic, prognostic and therapeutic implications involved. The need for an integrated harmonization of medical action for the pregnant patient and the embryo or foetus, and the characteristics of each gestational period, will determine the treatment protocol to be proposed and its limitations. For this reason, a group of experts appointed by participating scientific societies, which includes the Spanish Society of Medical Oncology (*Sociedad Española de Oncología Médica*—SEOM), the Spanish Association of Surgeons (*Asociación Española de Cirujanos*—AEC), the Spanish Society of Gynaecology and Obstetrics (*Sociedad Española de Ginecología y Obstetricia*—SEGO), the Spanish Society of Nuclear Medicine and Molecular Imaging (*Sociedad Española de Medicina Nuclear e Imagen Molecular*—SEMNIM), the Spanish Society of Oncological Radiotherapy (*Sociedad Española de Oncología Radioterápica*—SEOR) and the Spanish Society of Medical Radiology (*Sociedad Española de Radiología Médica*—SERAM), have worked together to establish consensus recommendations that allow the harmonization of the management and ultimately the optimization of the healthcare of pregnant patients with cancer.

## Diagnosis of cancer during pregnancy

**Fig. 1:**
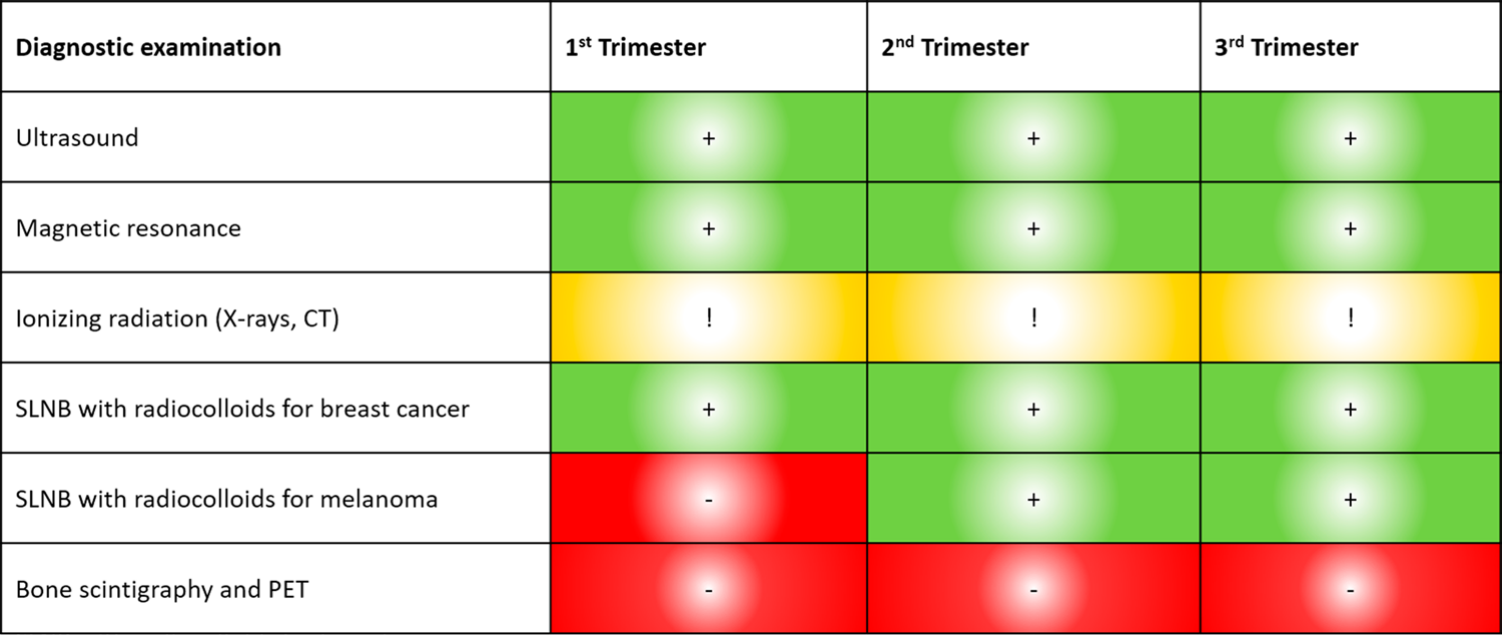
Recommendations for different diagnostic tests during pregnancy according to trimester. *SLNB* selective sentinel lymph node biopsy, *PET* positron emission tomography. Green: recommended; Yellow: depending on gestational age and tumour location; Red: not recommended

Figure 1 summarizes the recommendations for the different diagnostic tests to be performed during pregnancy according to trimester.

### Diagnosis by nuclear medicine

#### Sentinel lymph node biopsy (SLNB)

Sentinel lymph node biopsy (SLNB) is a standard technique in breast cancer staging and therefore should form part of the discussion and preparation of the diagnostic strategy of the multidisciplinary team responsible for the patient. The scientific evidence regarding SLNB in breast cancer during pregnancy is limited to cohort studies [4]; that is, there is no level 1 evidence, and the degree of the recommendations of the different guidelines is limited to C or D. Its use has not been validated in the *American Society of Clinical Oncology* (ASCO) guidelines due to the lack of data available in the literature [5]. The *National Comprehensive Cancer Network* (NCCN) *Clinical Practice Guidelines in Oncology* state that the decision must be made individually by each patient [6]. However, the *European Society for Medical Oncology* (ESMO) does not discourage its implementation in centres where it is a routine practice in non-pregnant patients [7]. Other guidelines recommend it when axillary ultrasound and suspicious lymph node biopsy have been negative [8, 9].

Lymphoscintigraphy and SLNB with ^99m^Tc-albumin nanocolloids do not cause significant uterine irradiation when optimized protocols are followed [10, 11]. Recommendations for minimizing foetal exposure are i) avoiding contact with other nuclear medicine patients by scheduling lymphoscintigraphy as the first procedure of the day and ii) reducing the time between nanocolloid injection and surgery. Thus, radiocolloids are considered safe [6, 8] when recommended as one-day protocols.

Regarding the efficacy of the procedure, in an international multicentre retrospective study with the largest cohort published to date (145 cN0 patients), the identification rate was high (99.3%), and the rate of axillary recurrence was very low (0.7%), which presupposes oncological safety for the mother; therefore, there is no reason to contraindicate SLNB during pregnancy [4].

Regarding SLNB in melanoma, results of a European survey showed great inconsistency regarding its performance in pregnancy [12]. However, SLNB with radiocolloids may be safely indicated after individual risk assessment [13]. The consensus is that it should be performed no earlier than the second trimester, in a one-day protocol (10–25 MBq ^99m^Tc) and with surgery scheduled immediately after lymphatic mapping [14, 15].

The main guidelines for gynaecological tumours during pregnancy do not recommend SLNB in cervical cancer. In the case of unifocal vulvar tumours less than 4 cm in size, due to the limited data available, SLNB should only be performed after assessing maternal safety and the risk of lymph node recurrence [16]. SLNB with vital blue dye is not recommended as anaphylactic reactions have been observed in 1–2% of patients [6, 7, 9, 11]. The safety and efficacy of SLNB by fluorescence with indocyanine green or with ferromagnetic particles have not been evaluated in pregnant patients [17].

Taking into account these data, this panel of experts recommends the following for pregnant patients:

- For breast cancer, SLNB should be offered with radiocolloids instead of axillary lymphadenectomy, provided that it is routinely performed in non-pregnant patients in the centre;
- For melanoma, SLNB with radiocolloids is considered safe and is recommended from the second trimester; and
- For gynaecological tumours, there are currently no data to establish a recommendation [18].

#### Imaging tests

The ESMO guidelines state that bone scintigraphy and positron emission tomography (PET) should be avoided during pregnancy [7]. Loibl et al. state that although radiotracers used in nuclear medicine and during PET probably do not produce exposure to radiation greater than 50 mGy, they cannot be recommended during pregnancy [11].

Some data from limited studies suggest that the dose of foetal radiation exposure is low with fluorodeoxyglucose (^18^F-FDG) PET and PET/magnetic resonance imaging (MRI), particularly during the later stages of pregnancy [19]. However, these data are insufficient to establish a recommendation for the use of PET for cancer staging during pregnancy.

### Diagnosis by radiology

Although the risk of radiation exposure during pregnancy is a common cause for concern, a missed or delayed diagnosis may pose an even greater threat to the woman and the foetus than any danger associated with ionizing radiation. The most effective way to limit radiation is (i) to use non-irradiated imaging modalities such as ultrasound, (ii) to justify all explorations and (iii) to use the lowest possible dose [20].

Ultrasound is the imaging technique of choice for pregnant women, although prolonged use of Doppler during the first trimester should be avoided. MRI is considered safe for the foetus in normal clinical use if safety standards are adhered to and ensuring that the magnetic field does not exceed 1.5 Tesla (T). The *American College of Radiology* (ACR) removed restrictions related to gestational age in a 2007 update [21].

The use of ionizing radiation, such as X-rays and computed tomography (CT), may produce biological alterations depending on gestational age and radiation dose. In the first 10-14 days of gestation, the only potential risk is abortion, although this has not been observed with the doses that are routinely administered (e.g., less than 1 mGy). From week 2 to week 15 of pregnancy, tests outside the abdomen (e.g., head and neck, thorax and extremities) do not pose a risk to the foetus because the only radiation that is delivered is scattered, very low and may be eliminated with standard precautions [22–24]. If testing procedures of the abdomen and pelvis are optimized, the dose of radiation received by the foetus will be well below any threshold that may induce developmental abnormalities. With doses less than 100 millisievert (mSv), there are no identifiable effects and termination of pregnancy is not justified [25]. With doses above 100 mSv, the risk of malformations is very low; with doses above 150 mSv, the risk of malformations during the development of the foetus increases and after week 15, the only potential risk is of cancer, induced with doses higher than 150 mSv. Foetal radiation doses up to 1 mGy are considered acceptable because it is the dose that a foetus receives as natural background radiation [26, 27].

Regarding iodinated contrast agents, no biological effects have been described following administration during pregnancy. The *US Food and Drug Administration* (FDA) considers them to be category B drugs; that is, no risk has been observed following their use in reproductive studies in animals. However, there have been no controlled studies in pregnant women. Because foetal thyroid development takes place during gestation, however, there is a potential risk of hypothyroidism. Therefore, the use of iodinated contrasts is recommended when no other examination is available, the information obtained is essential for both the mother and the foetus, and it is considered unwise to delay the test until after delivery [28].

The administration of gadolinium should be avoided during pregnancy because its effect when deposited in tissues is unknown. In addition, it is associated with rheumatological, inflammatory and infiltrative skin conditions [29].

During lactation, iodinated or gadolinium-based contrasts are considered safe for the baby because the average dose reached through the ingestion of breast milk is very small (0.01% of the dose received by the mother for gadolinium and 0.5% for iodine). In addition, only a small proportion that reaches the baby’s gastrointestinal tract is absorbed. The maximum peak of contrast agent concentration in breast milk occurs 5 h after injection and is undetectable at 12 h. The ACR in its *Manual on Contrast Media* states that, with the data currently available, continuing with breastfeeding is safe [30]. However, if after being informed, mothers do not wish to expose their babies to contrast agents, they should be instructed to discard breast milk for 24 h after contrast injection. In the case of premature babies, special attention should be paid because thyroid regulation is not yet mature; therefore, there is a risk of developing transient hypothyroidism [31].

It is mandatory that the medical team, of which the radiologist is a key member, reviews the indications for and frequency of radiological tests individually, as well as their risk–benefit ratios. In addition, the patient must be properly counselled and afterwards she must provide written informed consent. Although the risks to the foetus are small, it is important to ensure that radiation doses are kept as low as reasonably possible. A medical physicist must calculate the total dose that reaches the foetus and any potential associated risk if an unusually complex procedure requires high levels of radiation or if a patient has a medical condition that will require multiple exposures to radiation throughout the entire pregnancy.

## Surgical treatment of cancer during pregnancy

Standard anaesthetic procedures are considered safe during pregnancy. In general, surgical treatment should be avoided during the first trimester because of an observed association between surgery during weeks 3–5 of gestation and the appearance of neural tube defects in the foetus.

Abdominal surgery is considered safer in the second trimester, in which the risk of abortion is low and the size of the uterus allows surgical procedures. On the other hand, all procedures carried out after week 20 should be performed in a slightly left lateral decubitus position to avoid compression of the vena cava and to maintain cardiac output.

In addition, a laparoscopic approach is considered feasible and safe during pregnancy until weeks 26-28. To minimize the potential risk to the foetus of this approach, the following are required: (i) surgery duration less than 90 min, (ii) pneumoperitoneum with a maximum intra-abdominal pressure of 10–13 mmHg, (iii) open pneumoperitoneum and (iv) the presence of an expert surgeon.

In the postoperative period, certain analgesics such as paracetamol, non-steroidal anti-inflammatory drugs (NSAIDs), tramadol or morphine, and antiemetics (metoclopramide or ondansetron) may be prescribed. In the third trimester, NSAIDs have been associated with premature closure of the ductus arteriosus and possible pulmonary hypertension in the neonate in 50–80% of cases. In addition, it is advisable to administer thromboembolic prophylaxis with low molecular weight heparin because pregnancy is a procoagulant state [16].

### Breast cancer

Surgical treatment of breast cancer during pregnancy is a challenge, and published data are limited to retrospective studies and case series. In the past, it was erroneously thought that inducing an abortion could improve the prognosis of the patient but this assumption is no longer supported by current evidence. In general, surgical treatment is similar to that for a non-pregnant woman and, depending on the stage, lumpectomy or mastectomy may be performed. Surgery may be carried out in any trimester with little risk to the foetus. Breast reconstruction should be postponed until after delivery as well as post-mastectomy and post-lumpectomy irradiation [32–36]. The recommendations regarding SLNB are discussed in the section on diagnosis in this article.

### Cervical cancer

#### Treatment pathways and techniques

A laparoscopic approach for cervical cancer should be considered taking into account the laparotomic route, limited until 14–16 weeks of pregnancy. Regarding surgical techniques, there are safety data on simple trachelectomy but not on radical trachelectomy [37]. Lymphadenectomy is, in general, technically feasible until week 24.

Patients should receive adequate information about the risks to pregnancy, especially about the limitations imposed by pregnancy for certain treatments such as radiotherapy.

#### Procedures according to disease stage, with the aim of maintaining pregnancy

If cervical cancer is diagnosed at stage IA or IA1 before weeks 22–25 of gestation, conization alone is sufficient and safe during pregnancy although there is an increased risk of bleeding, which increases as pregnancy progresses. Simple trachelectomy is a safe option in these cases [37].

In the case of stage IA2, IB1, IB2 or IIA cervical cancer, if the patient is at a gestational age below 22 weeks, the first option is lymphadenectomy. In this situation (stage IIIC, with lymph node involvement confirmed by lymphadenectomy), if the pregnancy is continued, chemotherapy treatment with cisplatin should be offered. This strategy is considered safe for the foetus [39], as well as combinations of paclitaxel with platinum salts [38], although the preferred regimen is cisplatin monotherapy [38].

Any approach involving radiotherapy is incompatible with the intention of maintaining pregnancy. In the absence of suspicion of lymph node involvement, in stages IB2 and IIA, lymphadenectomy is not necessary, and chemotherapy may be chosen.

In general, in tumours smaller than 2 cm in patients in the third trimester, the option of maternal–foetal monitoring until foetal maturity may be considered, to be followed by surgery.

In locally advanced stages (IB3-IIA2-IIB), chemotherapy should be chosen. As indicated in the section on chemotherapy, cisplatin is a key drug in cervical cancer chemotherapy and may be safely administered without harm to the foetus—but not in the first trimester [39]. In these cases, the role of staging lymphadenectomy is unknown and is not recommended. At other more advanced stages, again chemotherapy is the treatment of choice when the choice is made to maintain pregnancy.

Regarding childbirth, elective caesarean section should generally be the preferred option. Following conization or trachelectomy during pregnancy, the risk of bleeding and cervical tearing is high. If a tumour has not been operated on during pregnancy, surgical treatment should be scheduled to occur at the same time as the caesarean section.

### Ovarian cancer

Regarding the aim of maintaining pregnancy, up until weeks 22-24, adequate surgical staging with preservation of the uterus should be performed. The need for contralateral adnexectomy is conditional upon gestational age and the condition of the ovary. In general, if gestational age compromises planned surgery or if it is not possible to perform adequate staging, then primary chemotherapy is indicated and cytoreductive surgery should be scheduled for after delivery. The same approach should be considered for cases of advanced ovarian cancer in which the aim is to continue the pregnancy.

### Colorectal cancer

The surgical treatment of colorectal cancer in pregnant patients depends on tumour location, stage, presentation and gestational age. If the diagnosis of cancer is made in early pregnancy, surgical resection of the tumour during pregnancy or termination of pregnancy followed by surgical resection should be considered. If the diagnosis of cancer is later in the pregnancy, early delivery may be induced if the gestational age of the foetus is acceptable for premature delivery, and then surgical treatment may be performed. In the case of rectal cancer, the surgical approach is very complicated during the last trimester and, although vaginal delivery is possible, caesarean section is recommended to avoid bleeding or the possibility of obstruction of the birth canal in the case of bulky tumours [39, 40].

## Radiotherapy for cancer during pregnancy

The administration of radiotherapy during pregnancy continues to be a topic of debate. In general, delaying radiotherapy is recommended until after delivery and it is contraindicated for tumours located in the pelvis because of the high risk of abortion and foetal harm [41].

Since the 1990s, technological improvements in modern radiotherapy such as three-dimensional conformal radiotherapy (3D-CRT), intensity-modulated radiation therapy (IMRT) and volumetric modulated arc therapy (VMAT), stereotactic radiotherapy and proton therapy have been introduced into clinical practice. The aim is to administer high doses of radiation to a tumour while reducing the risk of irradiating organs located in close proximity, thereby improving the effectiveness and tolerability of the treatment [42–45]. In addition, guided imaging techniques using cone-beam computed tomography (CBCT) have been developed to ensure the accurate administration of daily doses [46]. IMRT-VMAT limits the exposure of high doses to a more restricted volume, with the disadvantage of administering low doses to a larger volume of normal tissue. Taking this into account, the use of advanced radiotherapy techniques in pregnant women with cancer may increase the probability of short- and long-term adverse effects on the foetus; therefore, IMRT-VMAT should be used with caution in strictly selected patients [41].

The adverse effects of foetal irradiation vary according to the week of gestation and the irradiation dose. In the first or second trimester, when the distance between the foetus and the irradiation field is greater, there is a lower risk of foetal exposure; whilst in the third trimester, this distance is shortened, which may lead to greater foetal exposure to radiation. However, this distance may be large enough to safely irradiate brain, head and neck tumours [47–49].

The biological effects of radiotherapy on the embryo and foetus can be stochastic and deterministic. The stochastic effects are probabilistic; they do not have a threshold above which the effect appears, although the risk of appearance, but not severity, increases with the treatment dose. The deterministic effects are characterized by a cause–effect association; their severity increases with the dose, and there is a threshold dose below which an effect does not occur [47–49]. The dominant deterministic effect of irradiation prior to implantation is early embryo death [47, 49], although this is very low with doses < 100 mGy.

During organogenesis (weeks 2–7), the main effects of radiotherapy are congenital anomalies and growth retardation without mental retardation, although the risk increases with doses above 500 mGy [41]. The brain develops between weeks 8-15; therefore, in this phase, the potential effects of radiotherapy could be microcephaly and mental retardation. Mental function does not seem to decrease with doses < 100 mGy but does with doses > 100 mGy [41, 49, 50]. The incidence of mental retardation with doses between 100 and 499 mGy is estimated at 6% [41].

The effects of irradiation in the second trimester (weeks 16-25) are similar to those in the first trimester. The main risks include mental retardation, microcephaly, cataracts, sterility and cancer. The incidence of mental retardation is 2% with doses < 500 mGy [41]. The risk of sterility and neurological damage is lower in the second trimester than in the first trimester [41]. During the third trimester (week 25), the risk of mental retardation, impaired growth and microcephaly appears to be low, although cases with exposure < 500 mGy have been observed [41].

In summary, pregnant patients with cancer should be evaluated by a multidisciplinary team to determine the risks and benefits of administering radiotherapy, both for the woman and the foetus. There is no justification for not administering radiotherapy when doses to the foetus are < 100 mGy. Radiotherapy is contraindicated for tumours located in the pelvis. The potential role of IMRT-VMAT and stereotactic or proton radiotherapy should be limited to selected cases until more clinical evidence is available.

## Systemic treatment of cancer during pregnancy

As a general rule, systemic treatments (chemotherapy, hormone therapy, targeted therapies and immunotherapy) are contraindicated during the first trimester because they carry a high risk of malformation (up to 20%) and abortion and because there are no safety data available during this period.

Organogenesis occurs from the second week of gestation to the eighth week; therefore, chemotherapy could be administered from weeks 13 to 33. The classical chemotherapy regimens may be administered up to 3 weeks before the expected date of delivery to avoid coinciding with neutropenia or thrombopenia potentially caused by these treatments.

In general, in the last 2 trimesters of pregnancy, chemotherapy does not carry a significant risk of malformation to the foetus. There are, however, safety data for certain chemotherapy schedules during the second and third trimesters. In breast cancer, there is only one prospective series of pregnant women treated with the 5-fluorouracil, doxorubicin and cyclophosphamide (FAC) regimen [51]. Anthracyclines, such as doxorubicin, epirubicin, pegylated liposomal doxorubicin and non-pegylated liposomal doxorubicin, exhibit very low placental transfer; therefore, they are considered safe in the second and third trimesters [52]. In contrast, idarubicin has a low molecular weight, high lipophilicity and little affinity for placental P glycoprotein, and there are data on its teratogenicity, even in the second trimester [53]. The largest published case–control study of women with gestational breast cancer confirms that doxorubicin and epirubicin are agents without teratogenicity when administered in the second and third trimesters [54]; however, in other studies, a tendency towards prematurity and low birth weight has been observed [55]. Unlike idarubicin and daunorubicin, neither doxorubicin nor epirubicin produce acute or delayed cardiotoxicity. For this reason, the FAC, 5-fluorouracil, epirubicin and cyclophosphamide (FEC), doxorubicin and cyclophosphamide (AC) and epirubicin and cyclophosphamide (EC) regimens are the most frequently used in gestational breast cancer.

There is some experience with cytarabine in the treatment of leukaemia, more frequently in combination with doxorubicin than daunorubicin or idarubicin, due to its worse toxicity profile during pregnancy [56].

The administration of doxorubicin with ifosfamide has also been evaluated for the treatment of pregnant patients with soft tissue sarcomas. Nine patients were treated in the third trimester with doxorubicin (50 mg/m^2^, day (D) 1) and ifosfamide (2.5 g/m^2^/day D1– D2) with the corresponding dose of Mesna, without toxicity or complications for mothers or babies [57].

Anthracyclines in polychemotherapy regimens have been used in the second and third trimesters in patients with non-Hodgkin lymphoma (cyclophosphamide, doxorubicin, vincristine and prednisone [CHOP]) and in Hodgkin disease (doxorubicin, bleomycin, vinblastine and dexamethasone ABVD]).

With taxanes, the data are even more scarce and limited. In general, taxane administration is considered safe during the second and third trimesters. Although there are fewer data than for anthracyclines [58], studies in patients treated in the second and third trimesters do not demonstrate foetal malformation or excess toxicity [59]. Taxanes have a very low placental transfer, although as a general rule, weekly paclitaxel is preferred because of its more manageable toxicity profile.

Cisplatin has been used in the second trimester as a neoadjuvant treatment to delay radical hysterectomy in pregnant women diagnosed with cervical cancer. Unlike doxorubicin and epirubicin, in the 7 cases studied, significant concentrations of cisplatin were observed in cord blood and amniotic fluid. However, the published studies showed that the 8 babies of the treated mothers were born by caesarean section after week 32, and were completely healthy [60]. The combination of cisplatin with bleomycin is recommended for the treatment of germinal tumours without etoposide, due to its high haematological toxicity and an increased risk of delayed foetal growth. In ovarian cancer, carboplatin has been administered in combination with paclitaxel from the second trimester [61].

Anti-HER2 agents are contraindicated throughout pregnancy. In a series of 19 patients, treatment with trastuzumab was associated with oligohydramnios and anhydramnios in more than 70% of pregnancies [62], as was the doublet of trastuzumab and pertuzumab [63].

Anti-CD20 agents, such as rituximab, may be used from the second trimester, although they may cause some foetal immunodepletion; therefore, it is advisable to assess the risk–benefit ratio [64]. In contrast, interferon-alpha may be used in all three trimesters [65]. There are safety data, for both the mother and the baby, regarding the administration of imatinib for chronic myeloid leukaemia, from the second trimester [66]. Hormone therapy is contraindicated throughout pregnancy, and there are no safety data regarding tyrosine kinase inhibitors (TKIs) except imatinib [67, 68].

Regarding immunotherapy, anti-PD-1/PDL-1 antibodies are associated with a greater number of abortions in animal models, probably due to their role in the immunotolerance state of pregnancy. [69]. Although there are reports of isolated cases treated with nivolumab or nivolumab and ipilimumab, so far there is no acceptable level of safety to recommend their use during pregnancy. The use of bevacizumab during pregnancy is not recommended because of its antiangiogenic effect although there are very few published cases [70].

## Monitoring and termination of pregnancy in patients with cancer

Patients diagnosed with any type of cancer during pregnancy should be evaluated by a multidisciplinary tumour committee, including obstetric assessment, for decision-making. The wishes of the mother should prevail once she has been informed regarding the situation and the risks associated with pregnancy. In addition, these patients should be evaluated and treated in centres that have neonatal units, in which they can act appropriately in situations of premature delivery [34, 71, 72].

When cancer is diagnosed during pregnancy, both the effect of the pregnancy on the cancer and the possible effect of the cancer on the pregnancy should be taken into account [73–75].

### Effect of pregnancy on cancer

Pregnancy itself does not have a direct effect on the progression of a neoplasm but it can delay diagnosis because the symptomatology produced by the cancer can often be confused with symptoms of pregnancy, whereas, on other occasions, being pregnant may delay the carrying out of certain diagnostic tests. All this causes a delay in cancer diagnosis, and so it is not uncommon for pregnant women to be in more advanced stages at diagnosis.

In addition, pregnancy may affect decision-making, influence the treatment strategies proposed and, therefore, affect the outcome of the disease.

### Effect of cancer on pregnancy

In most cases, cancer will not influence the development of a pregnancy, especially regarding extragenital tumours. The most frequent risks are intrauterine foetal growth delays in advanced stages of pregnancy, low birth weight and iatrogenic prematurity due to early termination of pregnancy to carry out certain treatments.

Some treatments commonly used in cancer patients may have negative effects on pregnancy that should be considered before use. Thus, the safest treatments should be chosen, or others should be used after the end of pregnancy. Surgical procedures that do not affect the genital tract are usually well tolerated by both the mother and the foetus. In the case of genital tract tumours, surgical treatment is indicated in situations that may, immediately or later, threaten the life of the mother or that may subsequently affect the normal progress of the pregnancy or childbirth. It is necessary to decide the optimal time for surgery, provide prior adequate evaluation and information to the patient and determine whether surgery may be deferred until foetal viability is reached, thus avoiding extreme prematurity, unless there is danger to the life of the patient. A determining factor for making this type of decision is the time of pregnancy in which the diagnosis of cancer is made (i.e., first, second or third trimester) as well as the week of gestation in which it is found.

As indicated in the section on systemic treatment during pregnancy, in general, systemic treatments are contraindicated during the first trimester and should be administered only when necessary during the second and third trimesters. If delay is not possible, the choice of drug should consider the health of the foetus, and therefore, the therapeutic approach may vary (e.g., monochemotherapy instead of polychemotherapy).

In all cases in which a patient is treated for a tumour during pregnancy, the patient must be referred to, and followed up in, a high-risk obstetrics unit where appropriate controls should be performed and the decision to end the pregnancy can be made as a function of the obstetric conditions and the oncological situation of the patient, and assessed in a multidisciplinary manner. During pregnancy, exhaustive obstetric monitoring should be performed, with monthly visits to the office and obstetric ultrasound performed at least once a month, increasing the frequency of these if necessary. The use of obstetric ultrasound, monitoring foetal growth and well-being, and observing the amount of amniotic fluid and the placenta are procedures that should be reviewed with special attention.

In general, a foetus is viable from week 24 of gestation or when it weighs more than 500 grams. Starting at that week, if foetal extraction is necessary, intramuscular treatment with corticosteroids may be administered beforehand to aid the pulmonary maturity of the foetus (Table 2). At least 48 h before birth, magnesium sulphate should also be administered to achieve a neuroprotective effect. Whenever possible, attempts should be made to maintain pregnancy until week 37 or at least until week 35. If the situation does not permit, attempts should be made to maintain the pregnancy until week 32 and, if this is not possible, at least until week 28. Before week 28 (between weeks 24 and 28), sequelae for the foetus due to prematurity will be substantial; therefore, the risk–benefit balance should be carefully evaluated.

**Table 2:** Recommendations at the end of pregnancy for patients with cancer

	Corticosteroids	Neuroprotection (magnesium sulphate)	Route of delivery
Week ≥ 24–28	Yes	Yes	Caesarean section
Week > 28–32	Yes	Yes	Caesarean section
Week > 32–35	Yes	No	Allow vaginal delivery if there are favourable obstetric conditions
Week > 35–37	No	No	Allow vaginal delivery if there are favourable obstetric conditions

## Other considerations

### Providing information to patients on different treatment options

The diagnosis of cancer in a pregnant patient is a delicate situation that requires specialized management by a medical team, both during admission and when diagnosing, treating and informing the mother, her child and her family. Therefore, a multidisciplinary, highly specialized and motivated team of professionals is necessary to assess each case individually. In addition, it is important to have the technology and support of the necessary teams to manage the high complexity of this clinical situation, implement psychological support after the diagnosis and take into account the necessary ethical principles to provide the best possible care. Furthermore, it is necessary to open this field to the development of new research techniques and therapeutic approaches at the international level.

**Fig. 2:**
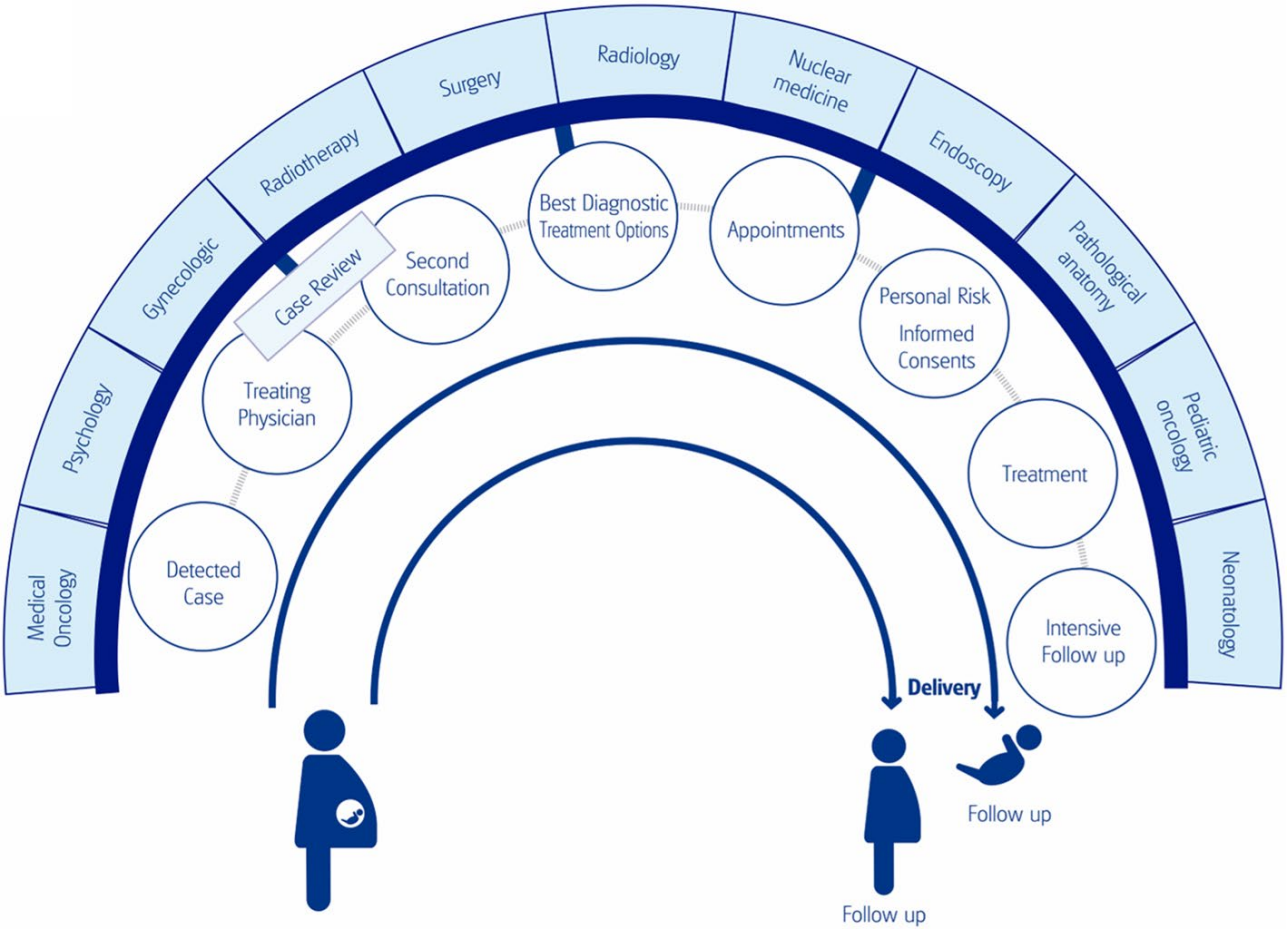
Flowchart of the multidisciplinary team involved during the informing, diagnosis and treatment of pregnant patients with cancer

Pregnant patients with a diagnosis of cancer require immediate multidisciplinary support, even in the initial communication of the diagnosis (Fig. 2). Medical professionals, at the individual level, often have reasonable doubts about the best way to act when informing, diagnosing, and treating these patients. Therefore, the formation of multidisciplinary tumour committees that, at least, include specialists in medical, paediatric, gynaecological and radiotherapy oncology, is strongly recommended, as well as specialists in gynaecology, psychology, surgery, radiology, nuclear medicine, neonatology, pathology and endoscopy.

The main objectives of these multidisciplinary teams are as follows:

1. To offer the optimal treatment for the mother and her child based on the cancer diagnosis, from the physical, psychological, social and spiritual viewpoint, as established by the World Health Organization (WHO);
2. To have a highly specialized team of professionals, continuously updated in the scientific, technical and ethical advances for the diagnosis and treatment of pregnant patients with cancer and their children;
3. To be a reference unit for a certain area or population of the country;
4. To have a clear impact on teaching and research tasks; and
5. To stimulate scientific progress, both in foetal diseases requiring intrauterine surgery and in medical treatments during pregnancy.

### Informed consent

When a doctor detects cancer in a pregnant patient in his practice, he must inform the patient that her case will be discussed within a multidisciplinary tumour committee that specializes in cancer during pregnancy, which will make a proposal with the best options for the diagnosis and treatment of her pathology. In a second consultation with the patient and her family, the options that are open should be explained, and doubts and questions that they pose should be resolved. Once an action plan is agreed, the medical team will provide the patient with the necessary appointments with the multidisciplinary team as a priority [76–79].

The patient must provide informed consent for the diagnostic and therapeutic procedures to which she has agreed. It is recommended that the patient be provided with an individual informed consent document that allows personalizing the risk of each of the planned diagnostic and treatment procedures (surgery, chemotherapy or radiotherapy). The documentation regarding the individualized informed consent signed by the patient should be included in her medical history [7, 42, 54, 80, 81].

## Conclusions

The most common form of cancer in pregnant women is breast cancer. In general, most diagnostic procedures may be performed in pregnant women without endangering the foetus, such as ultrasound, mammography with radioprotection and MRI without contrast. Radiation should be avoided, but if it is clinically justified for the benefit to the mother or foetus, there is no reason not to use it with adequate protection, taking into account that a delay in diagnosis may be worse for the patient, and always following the *as low as reasonably achievable* (ALARA) principle. In breast cancer, carrying the pregnancy to term does not mean a worse prognosis for the patient.

When cancer is detected in a pregnant woman, the week of gestation in which the diagnosis is made must be considered, as well as the characteristics of the tumour. It is strongly recommended that a multidisciplinary team assesses the situation and guides the patient and her family during the informing, diagnosis and treatment processes.

Chemotherapy may be administered starting at week 14 of gestation. In patients with breast cancer, treatment with doxorubicin should be the first option, and paclitaxel the second. Surgery may be carried out during pregnancy, but it is important to adjust the anaesthesia times as well as to carry out exhaustive control of postoperative pain, which may otherwise cause contractions that advance delivery.

The foetus should be monitored and be under the control of specialized obstetricians who are part of a multidisciplinary tumour committee who, if necessary, should perform an assessment before administering each chemotherapy cycle to the patient. Cancer does not affect the foetus, except in very rare cases of metastatic melanoma which carries a high burden of metastatic disease.

It is possible to treat cancer during pregnancy without sequelae for the baby. Data have been published on studies of women diagnosed with cancer during pregnancy whose children were subsequently born without sequelae.
